# The *ThSOS3* Gene Improves the Salt Tolerance of Transgenic *Tamarix hispida* and *Arabidopsis thaliana*

**DOI:** 10.3389/fpls.2020.597480

**Published:** 2021-01-15

**Authors:** Zhongyuan Liu, Qingjun Xie, Feifei Tang, Jing Wu, Wenfang Dong, Chao Wang, Caiqiu Gao

**Affiliations:** State Key Laboratory of Tree Genetics and Breeding, Northeast Forestry University, Harbin, China

**Keywords:** ROS-scavenging capability, salt stress, *Tamarix hispida*, *Arabidopsis thaliana*, *ThSOS*

## Abstract

The salt overly sensitive (SOS) signal transduction pathway is one of the most highly studied salt tolerance pathways in plants. However, the molecular mechanism of the salt stress response in *Tamarix hispida* has remained largely unclear. In this study, five *SOS* genes (*ThSOS1*–*ThSOS5*) from *T. hispida* were cloned and characterized. The expression levels of most *ThSOS* genes significantly changed after NaCl, PEG_6000_, and abscisic acid (ABA) treatment in at least one organ. Notably, the expression of *ThSOS3* was significantly downregulated after 6 h under salt stress. To further analyze *ThSOS3* function, *ThSOS3* overexpression and RNAi-mediated silencing were performed using a transient transformation system. Compared with controls, *ThSOS3*-overexpressing transgenic *T. hispida* plants exhibited greater reactive oxygen species (ROS)-scavenging capability and antioxidant enzyme activity, lower malondialdehyde (MDA) and H_2_O_2_ levels, and lower electrolyte leakage rates under salt stress. Similar results were obtained for physiological parameters in transgenic Arabidopsis, including H_2_O_2_ and MDA accumulation, superoxide dismutase (SOD) and peroxidase (POD) activity, and electrolyte leakage. In addition, transgenic Arabidopsis plants overexpressing *ThSOS3* displayed increased root growth and fresh weight gain under salt stress. Together, these data suggest that overexpression of *ThSOS3* confers salt stress tolerance on plants by enhancing antioxidant enzyme activity, improving ROS-scavenging capability, and decreasing the MDA content and lipid peroxidation of cell membranes. These results suggest that *ThSOS3* might play an important physiological role in salt tolerance in transgenic *T. hispida* plants. This study provides a foundation for further elucidation of salt tolerance mechanisms involving *ThSOS*s in *T. hispida*.

## Introduction

High salinity is a major adverse environmental factor affecting plant growth and development due to osmotic and ionic stress (El Mahi et al., 2019; Yang et al., 2019). Plants have evolved several mechanisms to respond to harsh environments and adjust their growth under high-salt conditions (Munns and Tester, 2008). Ca^2+^ is a ubiquitous secondary messenger that is involved in the signaling of various environmental and developmental stimuli (Hilleary et al., 2018; Srinivasan and Roberto, 2018). Rapid and significant changes in intracellular Ca^2+^ concentrations can occur in response to such stimuli, and these changes are sensed and decoded by Ca^2+^ sensors, including calmodulins, calmodulin-like proteins, calcineurin B-like proteins (CBLs), and Ca^2+^-dependent protein kinases (Kudla et al., 2010). Ca^2+^ sensors or Ca^2+^-binding proteins can sense transient Ca^2+^ changes and alter the protein phosphorylation and gene expression of related proteins or genes, thus enabling plants to survive stress conditions (Luan, 2009).

The *salt overly sensitive (SOS) 3* gene acts as a Ca^2+^ receptor in plants and is involved in Ca^2+^ signal-mediated stress responses. This gene, which encodes a calcineurin-like protein, belongs to the *CBL* gene family and is also known as *CBL4*. The *SOS3* gene was originally identified in the model plant Arabidopsis (Kamei et al., 2010). The *SOS3*-encoded protein contains EF-hand domains in its C-terminus and a myristylation site in its N-terminal region. Myristylation is important for recruitment of *SOS3* to the plasma membrane and for salt tolerance in plants (Quintero et al., 2002). SOS3 physically interacts with SOS2, a serine/threonine protein kinase. In the presence of Ca^2+^, *SOS3* activates the substrate phosphorylation activity of *SOS2*. The binding of *SOS3* to *SOS2* is mediated by the 21-amino acid (aa) FISL motif in *SOS2* and activates SOS1, a Na^+^/H^+^ antiporter, leading to Na^+^ efflux from the cytosol (Quintero et al., 2002; Nutan et al., 2018). Moreover, an *SOS2*–*SOS3* interaction in the SOS pathway has also been demonstrated using *sos3*/*sos2* double-mutant Arabidopsis (Guo et al., 2001). In addition to interacting with SOS3 at the plasma membrane, SOS2 has also been reported to interact with and thereby regulate the activity of several tonoplast-localized transporters, such as the Ca^2+^/H^+^ antiporter, vacuolar V-ATPase, and the Na^+^/H^+^ exchanger (Cheng et al., 2004; Batelli et al., 2007; Huertas et al., 2012).

*SOS3*s and *CIPK*s from different plant species have been found to function in different tissues in response to abiotic stress. For example, SOS2 and SOS3 specifically mediate salt stress signal transduction in Arabidopsis roots (Qiu et al., 2002; Zhang et al., 2017). *OsSOS2* and *OsSOS3* can coordinate to activate *OsSOS1* in yeast cells and can be exchanged with their Arabidopsis counterparts to form heterologous protein kinase modules that activate both *OsSOS1* and *AtSOS1* and suppress the salt sensitivity of *sos2* and *sos3* Arabidopsis mutants (Martínez-Atienza et al., 2007).

The *SOS3* gene has also been reported to be involved in plant salt stress responses. For example, in Arabidopsis, *SOS3* has been shown to play a unique role in these responses (Kamei et al., 2010). Notably, duplication of *SOS3* increases the Ca^2+^-mediated signaling capacity in *Eutrema* and confers increased salt tolerance on salt-sensitive Arabidopsis (Monihan et al., 2019). Overexpression of the *SOS3* gene in tobacco increases salt stress by causing exclusion of Na^+^ from the cytosol and retention of high K^+^ levels in the cytosol to re-establish ion homeostasis (Li et al., 2013). In *Populus trichocarpa*, *PtSOS1*, *PtSOS2*, and *PtSOS3* have been identified to cooperate in the activation of *PtSOS1*, thus conferring salt tolerance on *P. trichocarpa* (Tang et al., 2010).

*Tamarix hispida* is a woody halophyte species with excellent stress resistance. It can form natural forests in soil with a 1% salt content and is thus an excellent material for research on salt tolerance mechanisms and for cloning of salt tolerance genes. A search of *T. hispida* transcriptome libraries yielded 5 *ThSOS* genes. In this study, the 5 *ThSOS* genes (*ThSOS1*–*ThSOS5*) were cloned and characterized, and their expression levels under different abiotic stress conditions were analyzed by qRT-PCR. To further study *ThSOS3* function, appropriate vectors for *ThSOS3* overexpression and RNAi-mediated silencing were generated and transformed into *T. hispida*. The results showed that *ThSOS3* significantly improved salt tolerance in transgenic plants. The results of this investigation thus provide a candidate salt tolerance gene for forest genetic breeding.

## Materials and Methods

### Plant Growth and Stress Treatments

*T. hispida* seedlings (Turpan Desert Botanical Garden) were planted in pots containing a perlite/vermiculite/soil mixture (1:1:4 v/v) in a greenhouse (70–75% relative humidity; 14 h light/10 h dark photocycle, approximately 600 mmol m^–2^ s^–1^; 24°C). Uniformly developed 2 months old *T. hispida* seedlings were irrigated with a solution of 0.4 M NaCl, 20% (w/v) PEG_6000_ or 100 μM abscisic acid (ABA), and the tissues were harvested at 6, 12, 24, 48, and 72 h. Seedlings irrigated with fresh water were harvested at the corresponding time points as controls. Each treatment was repeated three times.

*Arabidopsis thaliana* Columbia (wild-type, WT) plants were used in this study. After vernalization, Arabidopsis seeds were sterilized for 3–5 min with 2.5% (v/v) sodium hypochlorite and washed three times with distilled water. The seeds were soaked on 1/2-strength Murashige and Skoog (MS) solid medium plates containing 0.6% agar. One-week-old seedlings were transferred from the plates to pots filled with vermiculite/soil/perlite (1:3:1) and grown in a greenhouse under a 16 h light/8 h dark photocycle, 70–75% relative humidity, 500 μmol m^–2^ s^–1^ light intensity, and a stable temperature of 22°C.

### Cloning and Sequence Analysis of *ThSOS*s

*ThSOS* genes were searched in the transcriptome library database and were further identified using conserved domain BLAST^1^. A phylogenetic tree was constructed with *T. hispida* SOS proteins and SOS homologs from other species using the neighbor-joining method in MEGA 5.0 via the bootstrap method with 1,000 replications (Tamura et al., 2011). Multiple sequence alignment was performed using ClustalX with a gap extension and gap opening penalties of 10 and 0.1, respectively (Thompson et al., 1997). The theoretical isoelectric point (pI) and molecular weight (MW) of each ThSOS protein were studied with the Expert Protein Analysis System (ExPASy) “compute pI/Mw” tool^2^. ThSOS subcellular localization was predicted using CELLO v.2.5^3^. The ∼2 kb upstream promoter region of each *ThSOS* gene was searched with the *T. hispida* genome database. The putative cis-acting elements of 3 *ThSOS* genes were analyzed with the Plant Cis-Acting Regulatory DNA Elements (PLACE) database^4^.

### RNA Extraction and qRT-PCR Analyses

Total RNA was extracted from *T. hispida* plants with a Plant RNeasy Extraction Kit (BioTeke, China), and first-strand cDNA was synthesized from 1 μg of purified RNA using a PrimeScript^TM^ RT Reagent Kit (TaKaRa, Beijing, China). qRT-PCR was performed on a qTOWER^3^ G (Analytik Jene AG, Germany) with the *Actin* (FJ618517) and β*-tubulin* (FJ618519) genes as internal controls. Each 20 μL reaction mixture contained 10 μL of SYBR-Green Real-time PCR Master Mix (Toyobo, Shanghai, China), specific primers (0.5 μM each), and 2 μL of cDNA template. Amplification was performed using the following cycling parameters: 94°C for 30 s; 45 cycles of 94°C for 12 s, 58°C for 30 s, and 72°C for 45 s; and then 82°C for 1 s for plate reading (Liu et al., 2018). The relative abundance was determined by the 2^–Δ^ ^Δ^ ^*Ct*^ method (Livak and Schmittgen, 2001). Three replicates were included for each sample (the primers used for qRT-PCR are shown in Table 1).

**TABLE 1:** Primers sequences used in this study.

Constructs	Forward and reverse primers (5′–3′)	
	
Genes	Primers used in real-time RT-PCR analysis	
*ThSOS1*	CTGATGCTGATCTGGATCCTAT	ATGCTAGACTGAAGAAATCGGT
*ThSOS2*	AGTAGAGGCCTTGTACGAGCT	ACCCAGTATGCCTCAGATCAT
*ThSOS3*	TGACGTTGATCCGATCAATTC	CCATAACAGGATCACATGCATAT
*ThSOS4*	CAATTTGCCTTATTCAGGAAT	CCACTATGCTCCCAACGATCT
*ThSOS5*	ATAGCCCACCATGGACGGCTT	ACCCTTGTGACTGAGAACCT
*Actin* (FJ618517)	AAACAATGGCTGATGCTG	ACAATACCGTGCTCAATAGG
β*-tubulin* (FJ618519)	GGAAGCCATAGAAAGACC	CAACAAATGTGGGATGCT

	**Primers used in constructing plant plasmids**	

pROKII-*ThSOS3*	GCTCTAGAATGGGCTGCTTCCATTCAAAG	GGTACCCCGTACTTCTGAATCTTCAACTT
pFGC5941-*ThSOS3*	*ThSOS3*-Sense-F:	*ThSOS3*-Sense-R:
	CATGCCATGGATGGGCTGCTTCCATTCAAAG	GCTCTAGATTATACTTCTGAATCTTCAACTTCCG
	*ThSOS3*-Anti-F: GCTCTAGAATGGGCTGCTTCCATTCAAAG	*ThSOS3*-Anti-R: CATGCCATGGTTATACTTCTGAATCTTCAACTTCCG

### Transient Expression of the *Thsos3* Gene in *T. hispida*

A 642 bp cDNA sequence of *ThSOS3* was amplified, cloned into the prokII vector with the CaMV 35S promoter, and named 35S::*ThSOS3*. A 200 bp truncated inverted-repeat cDNA sequence of *ThSOS3* was cloned into pFGC5941 flanking the CHSA intron to generate pFGC5941::*ThSOS3* and used to silence the expression of *ThSOS3* (the primers for vector construction are listed in Table 1).

*T. hispida* plants were transiently transformed according to the methods of Zhang et al. (2018). Three groups of transgenic *T. hispida* plants were generated by transient transformation: 35S::*ThSOS3* plants were generated to overexpress *ThSOS3* (OE), pFGC5941::*ThSOS3* plants were generated to silence the expression of *ThSOS3* (SE), and empty pROKII vector-transformed plants were generated as controls (Con). After growth for 12, 24, and 36 h under normal conditions or salt treatment, the relative abundance of *ThSOS3* in these transformed *T*. *hispida* plants was studied using qRT-PCR. Three biological replicates were included that contained at least 60 transformed seedlings.

### Stress Tolerance Analysis

Stably transformed Arabidopsis plants were generated by the floral dip method (Clough and Bent, 1998). Two T_3_ generation homozygous *ThSOS3* transgenic lines (OE1 and OE2) were selected to further evaluate stress tolerance. After vernalization, Arabidopsis seeds were sown on 1/2-strength MS medium and grown for 5 days prior to being transferred to 1/2-strength MS medium with or without 120 mM NaCl for 10–14 days. The root length and fresh weight were measured, and the seedlings were imaged. To assess salt tolerance in soil, seeds of two T_3_ generation homozygous transgenic lines (OE1 and OE2) of *ThSOS3* and the Con (WT) line were sown on 1/2 MS solid medium for 5–7 days and then transferred to soil. After 3 weeks of growth, the seedlings were irrigated with a 150 mM NaCl solution for 5 days and then imaged. The treatments were independently repeated at least three times. Each sample was analyzed and contained at least 15 transformed seedlings.

### Biochemical Staining

Hydrogen peroxide (H_2_O_2_) and superoxide (O^2–^) staining were performed by infiltration with 3-3-diaminobenzidine (DAB) or nitro blue tetrazolium (NBT) following the procedures described by Zhang et al. (2011). Evans blue staining was performed to investigate cell death, as described by Liu et al. (2018).

### Physiological Index Measurement of Transformed Plants

Transformed *T. hispida* plantlets were grown on 1/2 MS solid medium supplemented with 150 mM NaCl for 12–36 h. Four-week-old Arabidopsis seedlings were subjected to 150 mM NaCl for 5 days. After treatment, the seedlings were collected and subjected to physiological index analysis. Superoxide dismutase (SOD) and peroxidase (POD) activity and H_2_O_2_ content were measured using corresponding reagent kits (Nanjing Jiancheng Bioengineering Institute, China) according to the manufacturer’s instructions. The MDA content was determined according to the methods of Dhindsa et al. (1981). Electrolyte leakage was analyzed as described by Ben-Amor et al. (1999). Each sample contained at least nine harvested seedlings, and all of the experiments were repeated at least three times.

### Statistical Analyses

Statistical analyses were carried out using Excel software. The data were compared using Student’s *t*-test, and differences were considered significant if *P* < 0.05. ^∗^Represents a significant difference (*P* < 0.05), and ^∗∗^represents a very significant difference (*P* < 0.01).

## Results

### Gene Identification and Sequence Analysis of *ThSOS* Genes

In total, five candidate *SOS* genes were selected and identified. The 5 ThSOS proteins ranged from 213 to 1,165 aa in length (Table 2). Large variations were found in the theoretical pI values (ranging from 4.76 to 6.42) and the MW values (ranging from 22.42 to 128.83 kDa) of the proteins encoded by the five *ThSOS* genes. The prediction results showed that the five *ThSOS* genes were localized in the plasma membrane, cytoplasm, extracellular space, or chloroplasts (Table 2).

**TABLE 2:** Features of *ThSOS* genes in *T. hispida*.

Name	Locus	ORF (bp)	Introns	Protein length	Theoretical pI	Aliphatic index	Molecular weight (kD)	Localization predictions
*ThSOS1*	Unigene22889	3,498	11	1,165	6.42	103.24	128.83	Plasma membrane
*ThSOS2*	Unigene13265	1,371	2	456	6.29	92.08	51.44	Cytoplasmic
*ThSOS3*	Unigene1212	642	6	213	4.76	96.53	22.42	Cytoplasmic
*ThSOS4*	Unigene24293	927	11	308	6.22	105.1	33.58	Extracellular or Chloroplast
*ThSOS5*	Unigene1744	675	0	224	5.00	88.39	25.57	Cytoplasmic

**FIGURE 1:**
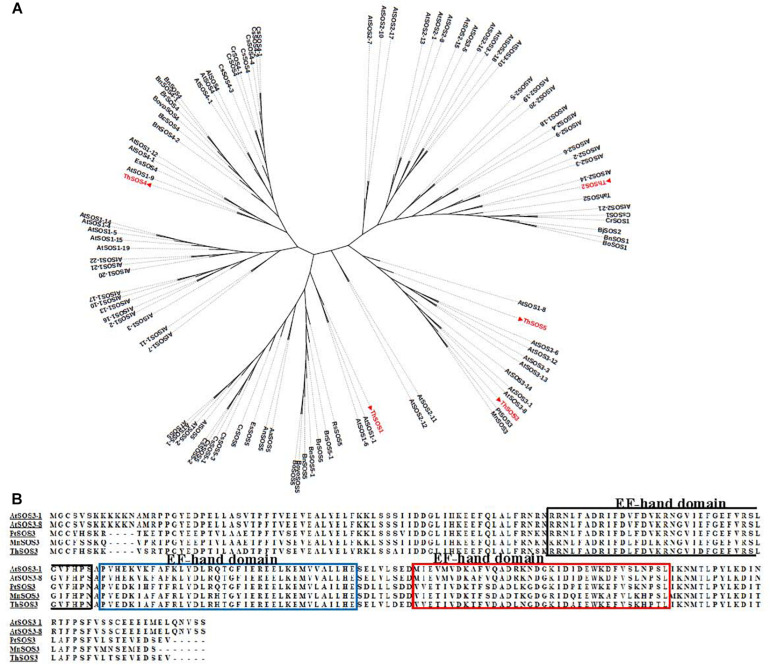
Phylogenetic and sequence analysis of ThSOS proteins. **(A)** Phylogenetic analysis of ThSOSs and other SOS proteins from different plant species. **(B)** Alignment of the ThSOS3 protein sequence with other plant SOS3 protein sequences. The sequences of SOS proteins were downloaded from the GenBank database, and their GenBank accession numbers are listed in Supplementary Table S1.

To determine the subclasses of the *ThSOS* genes, phylogenetic analysis was performed using the sequences of the ThSOS proteins and SOS proteins from other species (Figure 1A and Supplementary Table S1). The results revealed that *ThSOS1*, *ThSOS2*, and *ThSOS3* genes were closely related to the *SOS1*, *SOS2*, and *SOS3* subfamilies in Arabidopsis. *ThSOS3* belonged to the *CBL4* subfamily and clustered into the same clade as *PtrSOS3*, *MnSOS3*, and *AtSOS3.* Multiple sequence alignment analysis showed that *ThSOS3* was closely related to *PtrSOS3* (XP-002318422.1) from *P. trichocarpa*, *MnSOS3* (XP-010100753.1) from *Morus notabilis*, and *AtSOS3-1* (AT5G24270) from *A. thaliana* (Figure 1B).

### Characterization of *Cis*-Elements in *ThSOS* Gene Promoters

Numerous stress-related consensus cis-acting elements were detected, including an ABA-responsive element (ABRE), an antioxidant response element (ARE), and TC-rich repeats. Moreover, *ThSOS1* included 1 abiotic stress-related element, the MYB-binding site (MBS); nine hormone stress-related elements, such as the salicylic acid-responsive element (TCA element), methyl jasmonate (MeJA)-responsive element (CGTCA motif or TGACG motif), gibberellin-responsive element (TATC-box), and auxin-responsive element (TGA element); and 1 development-related endosperm expression element (GCN4 motif). *ThSOS3* contained an MBS and low temperature response (LTR) response elements consisting of five hormone stress-related elements, auxin-responsive elements (TGA elements or AuxRR core elements), and salicylic acid-responsive elements (TCA elements). Similarly, *ThSOS4* contained many abiotic and hormone stress-related elements, as shown in Supplementary Figure S1.

### Expression of *ThSOS* Genes Under Abiotic Stress and ABA Treatment

To analyze the relative abundance of *ThSOS* genes, the expression profiles of the 5 *ThSOS* genes were measured under different stresses (NaCl or PEG_6000_ exposure) or hormone treatment (ABA) using qRT-PCR.

**FIGURE 2:**
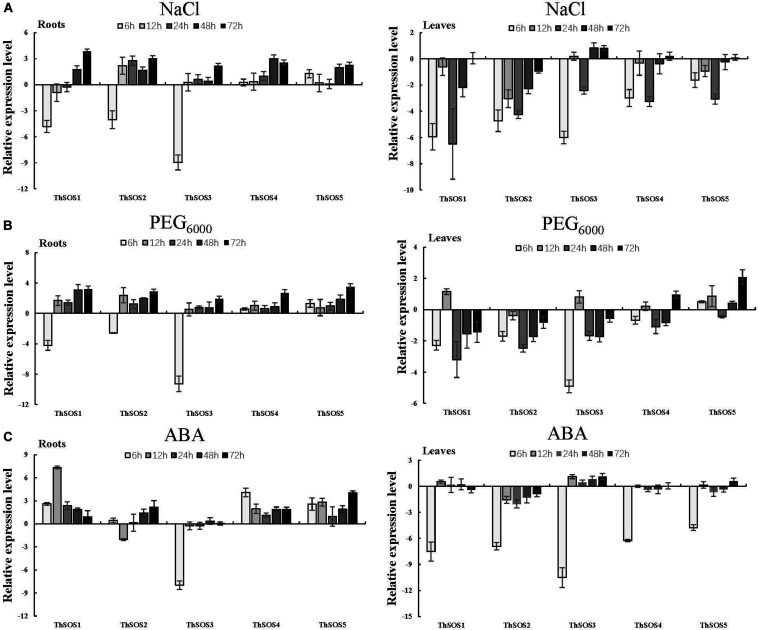
Expression analysis of the 5 *ThSOS* genes in the roots and leaves in response to abiotic stresses (NaCl and PEG_6000_ exposure) and hormone (ABA) treatment. **(A)** 0.4 M NaCl. **(B)** 20% (w/v) PEG_6000_. **(C)** 100 μM ABA. All relative transcription levels were log2-transformed. The error bars were obtained from multiple replicates of qRT-PCR.

In roots under NaCl stress, the expression of most *ThSOS* genes was upregulated. Notably, *ThSOS4* and *ThSOS5* exhibited upregulated expression at all stress time points. The highest expression levels of *ThSOS4* and *ThSOS5* were 8.02- and 4.86-fold higher than control levels, respectively. The other three *ThSOS* genes, *ThSOS1*, *ThSOS2*, and *ThSOS3*, were downregulated at the initial stress time point and upregulated at later stages. The lowest expression levels of the three *ThSOS* genes in the roots all occurred at 6 h. The expression levels of *ThSOS1*, *ThSOS2*, and *ThSOS3* at this time point were 3.55, 6.11, and 0.20% of control levels, respectively. These results indicate that these 3 genes can respond rapidly to salt stress in *T. hispida* roots. In leaves, *ThSOS* gene expression was mainly downregulated during the stress period. *ThSOS2* and *ThSOS3* reached their lowest expression levels (3.78 and 1.56% of baseline levels, respectively) in the control plants at 6 h. The relative abundance of *ThSOS1*, *ThSOS4*, and *ThSOS5* was similar to that of *ThSOS2* and *ThSOS3*, but the lowest expression levels were achieved at 24 h (Figure 2A).

Under PEG_6000_ stress, most of the *ThSOS* genes were significantly upregulated, and all *ThSOS* genes achieved their highest expression levels at 72 h. Interestingly, the expression levels of *ThSOS1*, *ThSOS2*, and *ThSOS3* in the roots were significantly downregulated after 6 h of PEG_6000_ stress (5.39, 16.67, and 0.16% of the control levels, respectively). In leaves, the expression levels of the *ThSOS1*, *ThSOS2*, and *ThSOS3* genes were mainly downregulated throughout the stress period, and *ThSOS1* and *ThSOS2* achieved their lowest expression levels at 24 h. However, *ThSOS3* reached its lowest expression level during the early stage of stress (6 h). In contrast to the gene expression patterns of these three *ThSOS* genes, the relative expression of *ThSOS5* was significantly upregulated at almost all stress points (in addition to 24 h) and peaked at 72 h. The expression of *ThSOS4* did not change significantly under PEG6000 stress (Figure 2B).

Under ABA stress, the relative abundance of *ThSOS1*, *ThSOS4*, and *ThSOS5* was significantly upregulated in the roots. The most strongly upregulated gene was *ThSOS1*; its expression peaked at levels 161.28-fold higher than control levels at 12 h. The relative expression of *ThSOS2* was mainly upregulated except at 12 h, when its expression was only 24.3% of the control level. However, *ThSOS3* expression was clearly downregulated at 6 h (0.4% of the control level) and showed no significant changes at any other stress time points. In leaves, no significant changes were found in the expression of any of the *ThSOS* genes except at 6 h. All the *ThSOS* genes (*ThSOS1*, *ThSOS2*, *ThSOS3*, *ThSOS4*, and *ThSOS5*) reached their lowest expression levels under ABA stress at 6 h (0.55, 0.83, 0.07, 1.31, and 3.17% of the control levels, respectively) (Figure 2C).

### Transient Expression of *ThSOS3* in *T. hispida*

**FIGURE 3:**
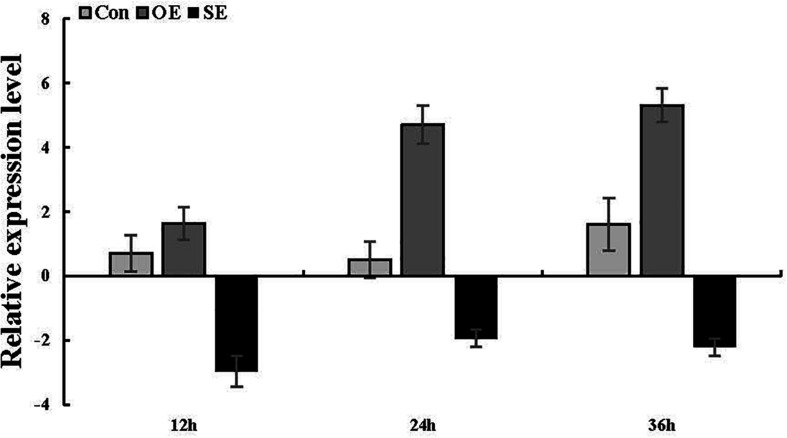
*ThSOS3* transcript levels in *T. hispida* plants with transient overexpression or knockdown of *ThSOS3*. The expression data were log2-transformed. Two-month-old *T. hispida* plants were transiently transformed with empty pROKII, 35S::*SOS3* or pFGC::*SOS3*. After transformation for 36 h, *T. hispida* plants were treated with 150 mM NaCl for 12, 24, or 36 h, and the expression of *ThSOS3* was determined. OE, *ThSOS3* overexpression; SE, *ThSOS3* RNAi; Con, pROKII vector control.

To ascertain whether the *ThSOS3* gene was successfully transiently overexpressed and suppressed in *T. hispida*, the *ThSOS3* transcript levels in Con, OE, and SE plants were examined by qRT-PCR. Compared with that in Con plants, the *ThSOS3* expression in OE plants was significantly increased under salt stress conditions, while that in SE plants was significantly decreased (Figure 3), indicating that the gain and loss of function of *ThSOS3* in *T. hispida* plants were successfully achieved.

### *ThSOS3* Confers Salt Stress Tolerance on Transgenic Plants

**FIGURE 4:**
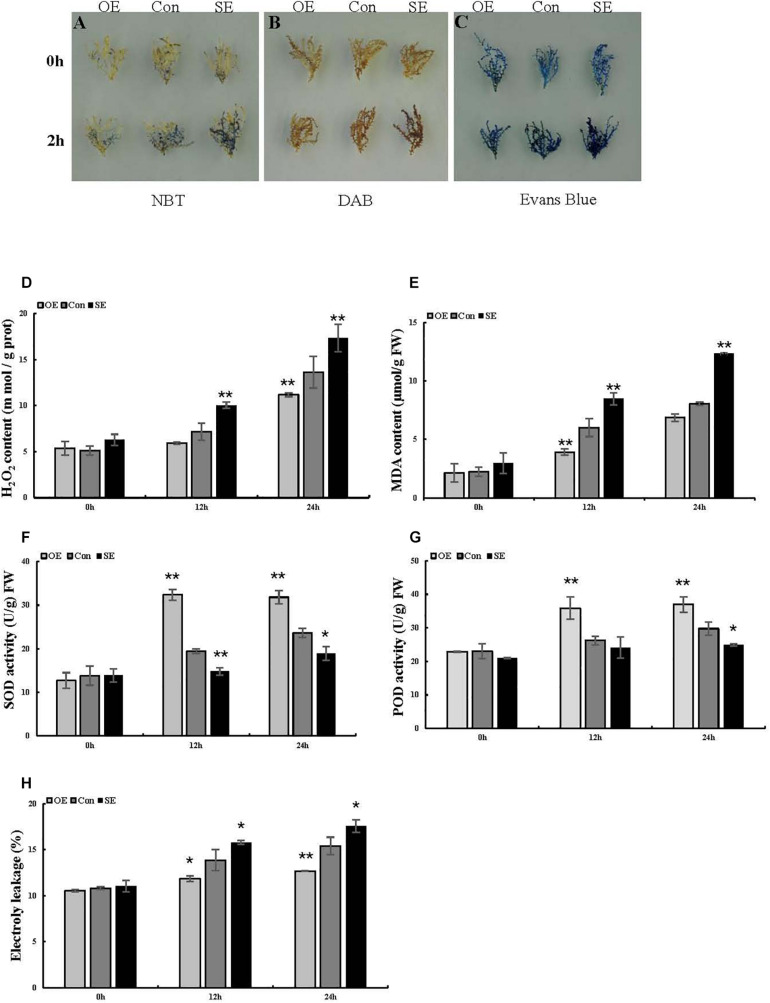
Analysis of ROS-scavenging capability and cell death in *T. hispida* plants with overexpression or RNAi-mediated knockdown of *ThSOS3*. **(A)** NBT and **(B)** DAB staining were performed to detect O^2–^ and H_2_O_2_, respectively. **(C)** Evans blue staining was used to analyze cell death. Young branches from transformed *T. hispida* plants treated with 150 mM NaCl for 2 h were used for DAB, NBT, and Evans blue staining. **(D–H)** Analysis of H_2_O_2_ and MDA content, SOD and POD activity, and electrolyte leakage in three different transgenic *T. hispida* plants. Transformed *T. hispida* plantlets grown on 1/2 MS solid medium supplemented with 150 mM NaCl for 24 h were used to measure the H_2_O_2_ **(D)** and MDA **(E)** content, SOD **(F),** and POD **(G)** activity, and electrolyte leakage **(H)**. *Represents a significant difference (*P* < 0.05). **Represents a very significant difference (*P* < 0.01).

To preliminarily explore the function of the *ThSOS3* gene, DAB staining and NBT staining were performed, and the related physiological indexes of three differently transformed *T. hispida* plants were studied. The reactive oxygen species (ROS) accumulation levels in OE, SE, and Con plants before and after abiotic stress were determined by DAB and NBT staining. Under salt stress, the staining intensity in OE plants was lower than that in Con plants, while that in SE plants was higher than that in Con plants (Figures 4A,B). Additionally, H_2_O_2_ and MDA levels were measured in different transgenic *T. hispida*. The results failed to demonstrate differences in H_2_O_2_ and MDA levels among the three transiently transgenic plants under normal conditions. However, under salt stress, SE plants showed the highest H_2_O_2_ and MDA levels, followed by Con plants; the OE plants had the lowest H_2_O_2_ and MDA levels. The levels of H_2_O_2_ and MDA in SE plants were 1.27 and 1.53 times those in Con plants, respectively. However, the H_2_O_2_ and MDA levels in OE plants were the lowest at only 82.02 and 85.2% of those in Con plants, respectively, at 24 h (Figures 4D,E).

**FIGURE 5:**
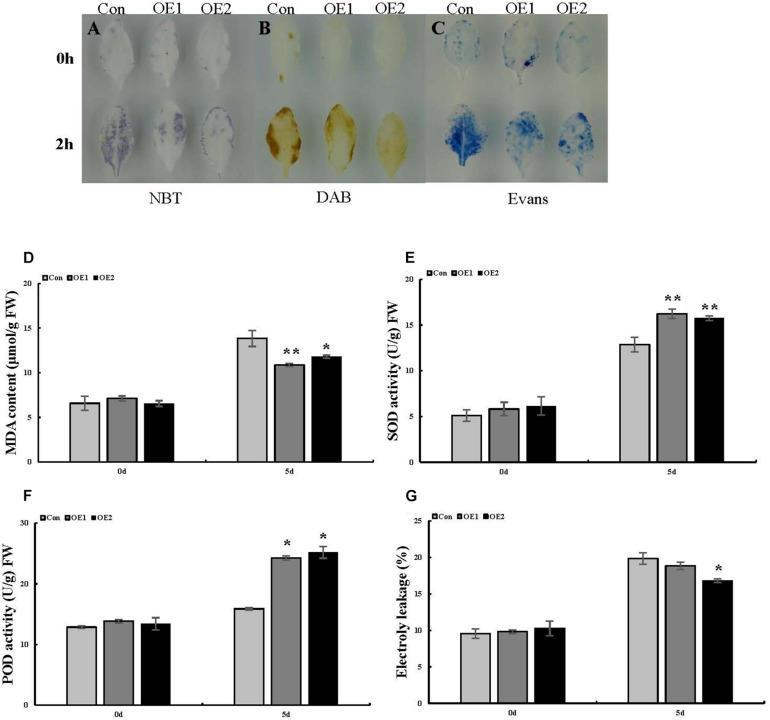
Analysis of ROS-scavenging capability and cell death in Arabidopsis plants overexpressing *ThSOS3*. **(A)** NBT, **(B)** DAB, and **(C)** Evans blue staining were performed. Leaves from *ThSOS3*-transformed and Con (WT Arabidopsis) plants treated with 150 mM NaCl for 24 h were used for histochemical staining. **(D–F)** Analysis of MDA content, SOD and POD activity, and electrolyte leakage in transgenic and Con (WT Arabidopsis) plants. Four-week-old Arabidopsis seedlings subjected to 150 mM NaCl for 24 h were used to detect the MDA **(D)** content, SOD **(E)** and POD **(F)** activity, and electrolyte leakage **(G)**. *Represents a significant difference (*P* < 0.05). **Represents a very significant difference (*P* < 0.01).

**FIGURE 6:**
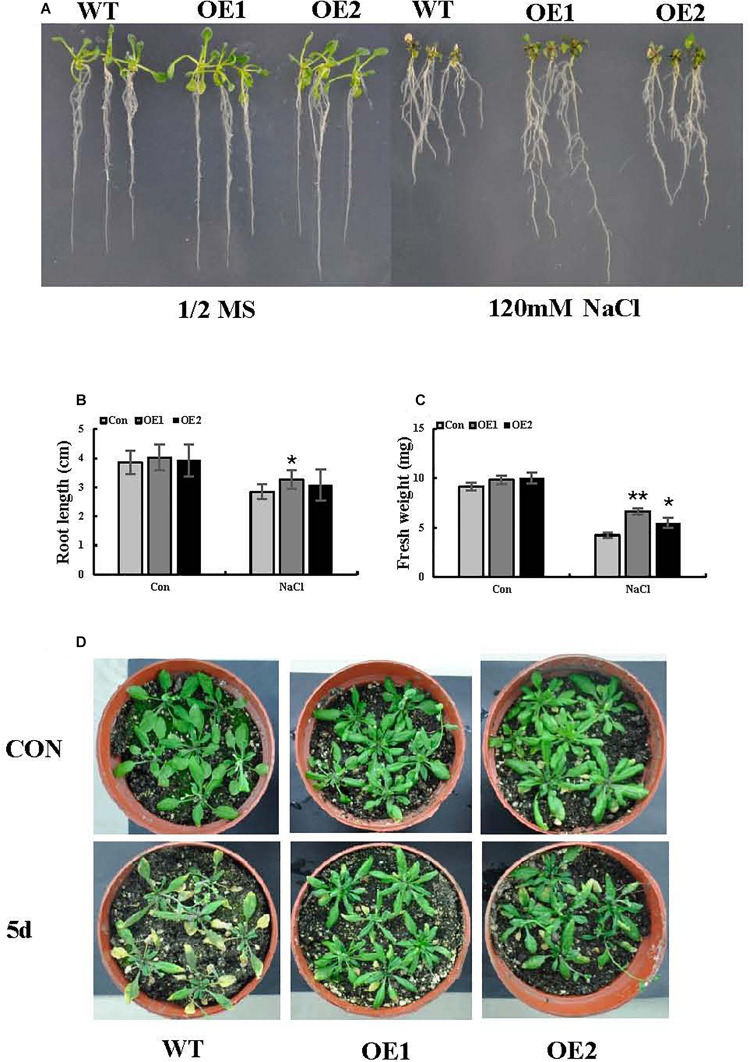
Salt stress tolerance associated with *ThSOS3*. **(A)** Growth comparison between OE and WT plants. Arabidopsis plants grown on 1/2-strength MS medium (control) and 1/2-strength MS medium supplied with 120 mM NaCl were used for growth analysis. **(B)** Root length and **(C)** fresh weight were also analyzed in at least 30 seedlings under each treatment. **(D)** Comparison of growth phenotypes between OE and WT Arabidopsis lines grown in soil. The plants were treated with 150 mM NaCl for 5 days for analysis. Plants grown under normal conditions were used as controls. *Represents a significant difference (*P* < 0.05). **Represents a very significant difference (*P* < 0.01).

To better interpret the results of transient expression of *ThSOS3* in *T. hispida*, *ThSOS3* was overexpressed in Arabidopsis. Two independent T_3_ homozygous transgenic lines (OE1 and OE2) overexpressing *ThSOS3* were selected and studied. Under salt treatment, the H_2_O_2_ and MDA levels in both OE lines were lower than those in the WT line, although the lines had similar H_2_O_2_ and MDA levels under normal conditions (Figures 5A,B,D). In addition, the phenotype, fresh weight, and root length were not different between the transgenic and WT plants under normal conditions. However, under salt treatment, the OE1 and OE2 plants presented significantly higher fresh weights and root lengths than the WT plants (Figures 6A–D).

### *ThSOS3* Improves ROS-Scavenging Capability

The antioxidant enzymes SOD and POD are the two most important ROS-scavenging enzymes influencing cellular ROS levels. Thus, we further studied POD and SOD activity. Under normal growth conditions, there were no obvious differences in activity levels between Con and transgenic *T. hispida*. However, SOD and POD activity levels were significantly increased in OE plants under salt stress. At 24 h, the activity levels of SOD and POD in OE plants were 1.35 and 1.24 times those in Con plants, while the activity levels in SE plants were only 80 and 83.73% of those in Con plants, respectively (Figures 4F,G).

Similarly, in Arabidopsis, SOD and POD activity did not obviously differ among the studied lines in the absence of stress. Under salt stress, the two OE lines showed higher antioxidant enzyme (SOD and POD) activity than the WT line (Figures 5E,F), consistent with the results obtained in *T. hispida*.

### Cell Death and Electrolyte Leakage Analysis

Evans blue staining was used to assess cell membrane damage on the basis of the intensity of the staining under salt stress. Compared with the Con plants, the OE plants presented light-blue puncta with smaller areas under salt stress, while SE plants presented larger staining areas (Figure 4C). We then measured electrolyte leakage. Electrolyte leakage did not significantly differ among the three differently transformed *T. hispida* plants under normal conditions. Under salt stress, the relative electrolyte leakage rates of SE plants were the highest at 24 h at 1.14 times those of Con plants, while the rates of the OE plants were 0.82 times those of the Con plants (Figure 4H). Moreover, we detected changes in the levels of corresponding physiological indicators in Arabidopsis, which were consistent with the changes in *T. hispida* (Figures 5C,G).

## Discussion

The *SOS* gene plays an important role in plants, and its function has been studied in *A. thaliana* (Qiu et al., 2004; Yang et al., 2009; Wang et al., 2019), *Nicotiana tabacum* (Yue et al., 2012), *Oryza sativa* (El Mahi et al., 2019), *Gossypium raimondii* (Che et al., 2019), *Lycopersicon esculentum* (Olias et al., 2009), *Zea mays* (Zörb et al., 2005), and *P. trichocarpa* (Tang et al., 2010; Zhou et al., 2014). However, few studies have investigated the salt tolerance function of *ThSOS* in *T. hispida*.

In our study, five monomorphic and intact *ThSOS* genes were selected. An unrooted phylogenetic tree and multiple sequence alignment analysis showed that *ThSOS3* shared 85.92, 84, and 70% identity with *PtrSOS3*, *MnSOS3*, and *AtSOS3-1*, respectively. It has been reported that *AtSOS3* and *PtrSOS3* enhance salt tolerance in Arabidopsis and *P. trichocarpa* (Quan et al., 2007; Tang et al., 2010). The relative abundance of most *ThSOS* genes in *T. hispida* was significantly changed under NaCl, PEG_6000_, and ABA stresses. Notably, *ThSOS3* expression was significantly downregulated under salt stress at 6 h. SOS3 is a Ca^2+^-regulated upstream regulatory protein of the SOS pathway and plays important roles in plant salt stress response pathways (Yang et al., 2009). Kim et al. (2013) confirmed that *AtSOS3* expression is strongly induced by NaCl treatment. In addition, overexpression of *LeSOS3-1* enhances salt stress tolerance in tobacco by regulating stress-associated physiological changes, such as by enhancing ROS-scavenging capability and maintaining K^+^/Na^+^ homeostasis.

Regulatory elements in promoter sequences are essential for the temporal, spatial, and cell type-specific control of gene expression (Jiang et al., 2014). Previous studies have shown that many abiotic and hormone stress-related elements are present in *ThSOS* gene promoters. As shown in Supplementary Figure S1, ABREs, AREs, and TC-rich repeats were found in the promoters of three *ThSOS* genes. The LTR element and MBS element were found in the promoter of the *ThSOS3* gene. *ThSOS3* also contained 4 hormone stress-related elements (a TGA element, a TCA element, an ABRE, and an AuxRR-core element). This result indicates that *ThSOS* might be involved in stress responses (to abiotic stress and hormone treatment) as well as in plant development. Moreover, previous studies have shown that *OSBZ8* mediates salt and dehydration stress tolerance by binding to the ABRE motif (Narusaka et al., 2003), and *AtMYB44* inhibits oxidative damage and hypersensitivity to abiotic stresses by binding to MBSs to activate the expression of related downstream genes (Persak and Pitzschke, 2014).

When the results of the analysis of cis-elements in *ThSOS* gene promoters and the phylogenetic analysis were combined, *ThSOS3* was shown to contain abundant abiotic and hormone stress-related elements and to be closely related to *AtSOS3* and *PtrSOS3*. Therefore, we predict that *ThSOS3* might also play roles in responses to salt stress.

Plants produce high levels of ROS in adverse environments. ROS act as signaling molecules to control several physiological processes (Liu et al., 2018). Baxter et al. (2013) found that H_2_O_2_ and O^2–^ signaling networks are involved in responses to abiotic stimuli. Under salt stress, the staining intensity was lower in OE plants and higher in SE plants than in Con plants. Furthermore, the H_2_O_2_ levels were consistent with the DAB and NBT staining results in transgenic *T. hispida* plants. The results showed that overexpression of *ThSOS3* resulted in the lowest H_2_O_2_ and MDA accumulation. Conversely, compared to *ThSOS3* overexpression, RNAi silencing induced the opposite physiological changes among the three differently transformed *T. hispida* plants. Next, Evans blue staining was performed to assess cell death in *T. hispida* plants under salt stress. The results indicated that compared with Con plants under salt stress, OE plants under salt stress presented light blue puncta with smaller areas, while SE plants showed the opposite results. We then measured electrolyte leakage, which did not significantly differ among the three differently transformed *T. hispida* plants under normal conditions. Under salt stress, the relative electrolyte leakage rates of SE plants were the highest at 24 h at 1.14 times those of Con plants; moreover, those of the OE plants were 0.82-fold those of the Con plants. The electrolyte leakage assay further confirmed the Evans blue staining results. Similar to our study, a previous study revealed that overexpression of the wheat *TaAQP8* gene can confer salt tolerance on transgenic tobacco plants by maintaining ionic balance, reducing H_2_O_2_ accumulation and reducing membrane damage (Hu et al., 2012). In addition, *ThWRKY4* can improve tolerance to salt and ABA treatment by increasing SOD and POD activity, decreasing O^2^ and H_2_O_2_ levels, reducing electrolyte leakage, preventing chlorophyll loss, and protecting cells from death (Zheng et al., 2013).

As ROS levels were significantly altered, we further studied the activity of POD and SOD, which are the two most important ROS-scavenging enzymes. In the absence of stress conditions, the activity levels of these enzymes did not significantly differ among the Con, OE, and SE plants. However, SOD and POD activity levels were significantly increased in OE plants under salt stress. At 24 h, the activity levels of SOD and POD in OE plants were 1.35 and 1.24 times those in Con plants, while the levels in SE plants were 80 and 83.73% of Con plants, respectively. As in our study, *ThZFP1* was shown to enhance salt and osmotic stress tolerance in a previous study by positively regulating proline accumulation and SOD and POD activity (Zang et al., 2015). In addition, *ThNAC7* has been found to induce the transcription of genes associated with stress tolerance to enhance salt and osmotic stress tolerance by increasing osmotic potential and enhancing ROS scavenging (He et al., 2019). In summary, the physiological indicator results suggest that *ThSOS3* confers salt stress tolerance by increasing the activity of antioxidant enzymes (SOD and POD), reducing ROS accumulation, and decreasing the MDA content and lipid peroxidation of cell membranes.

## Conclusion

The *SOS* gene plays important roles in responses to salt stress. However, few studies have evaluated the roles of *ThSOSs* in salt tolerance in *T. hispida*. In this study, five *ThSOS* genes were cloned and identified. Their expression patterns in response to different abiotic stresses (NaCl and PEG_6000_ exposure) and hormone (ABA) stress were analyzed using qRT-PCR. The expression levels of most *ThSOS* genes were significantly altered under NaCl, PEG_6000_, and ABA treatment in at least one organ. Notably, *ThSOS3* expression was significantly downregulated under salt stress at 6 h. Furthermore, the role of *ThSOS3* in salt tolerance was studied. The results showed that overexpression of *ThSOS3* confers salt stress tolerance on *T. hispida* by enhancing antioxidant enzyme activity, improving ROS-scavenging capability and decreasing the MDA content and lipid peroxidation of cell membranes. This study provides a foundation for further elucidation of salt tolerance mechanisms involving *ThSOS*s in *T. hispida*. However, the molecular mechanism by which *ThSOS3* confers salt stress tolerance on *T. hispida* is unclear. Future studies should focus on *ThSOS3* mechanisms under salt stress.

## Data Availability Statement

The raw data supporting the conclusions of this article will be made available by the authors, without undue reservation.

## Author Contributions

CG designed the research. ZL and FT conducted the experiments and performed data analysis. ZL and QX wrote the manuscript. JW and WD performed data analysis. CW and CG revised the manuscript. All authors read and approved the manuscript.

## Conflict of Interest

The authors declare that the research was conducted in the absence of any commercial or financial relationships that could be construed as a potential conflict of interest.

## Footnotes

1 https://blast.ncbi.nlm.nih.gov/Blast.cgi?PROGRAM=blastx&PAGE_TYPE=BlastSearch&LINK_LOC=blasthome

2 http://www.expasy.org/tools/protparam.html

3 http://cello.life.nctu.edu.tw/

4 http://bioinformatics.psb.ugent.be/webtools/plantcare/html/

## Supplementary Material

Supplementary Figure 1 **(A)** Locations of cis-elements in the *ThSOS* gene promoters. **(B)** Descriptions of the cis-elements.

Supplementary Table 1 Classification of SOS genes in different species.
