# Supplementary material for: The ThSOS3 Gene Improves the Salt Tolerance of Transgenic Tamarix hispida and Arabidopsis thaliana
Source: Front Plant Sci. 2021 Jan 15;11:597480. doi: 10.3389/fpls.2020.597480 (PMC7848111; doi:10.3389/fpls.2020.597480)
Supplement: Supplementary Figure 1 — (A) Locations of cis-elements in the ThSOS gene promoters. (B) Descriptions of the cis-elements. [file Image_1.pdf]

A

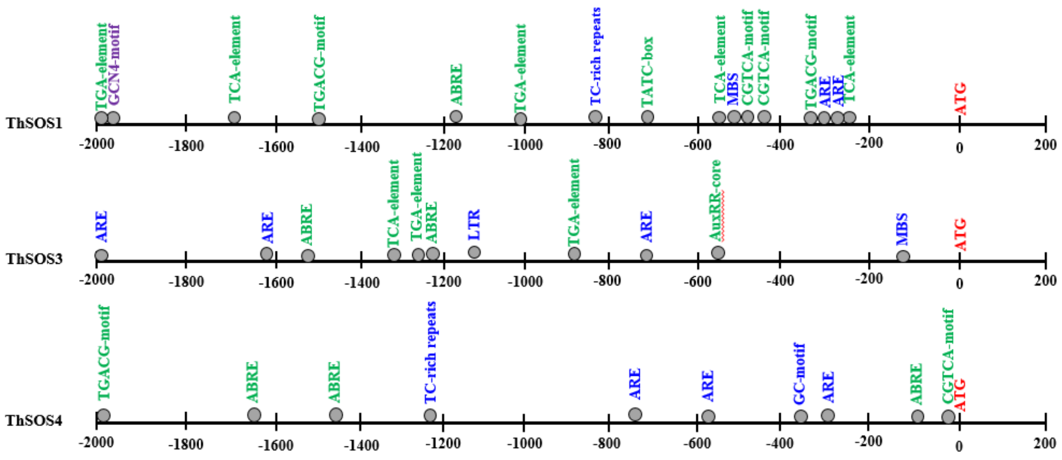

B

Class	Element	Description
Hormone	ABRE	cis-acting element involved in the abscisic acid responsiveness
	AuxRR-core	cis-acting regulatory element involved in auxin responsiveness
	CGTCA-motif	cis-acting regulatory element involved in the MeJA-responsiveness
	TATC-box	cis-acting element involved in gibberellin-responsiveness
	TCA-element	cis-acting element involved in salicylic acid responsiveness
	TGACG-motif	cis-acting regulatory element involved in the MeJA-responsiveness
	TGA-element	auxin-responsive element
Stress	ARE	cis-acting regulatory element essential for the anaerobic induction
	MBS	MYB binding site involved in drought-inducibility
	LTR	cis-acting element involved in low-temperature responsiveness
	GC-motif	enhancer-like element involved in anoxic specific inducibility
	TC-rich repeats	cis-acting element involved in defense and stress responsiveness
Development	GCN4-motif	cis-regulatory element involved in endosperm expression

Fig. S1
