# Supplementary material for: The ThSOS3 Gene Improves the Salt Tolerance of Transgenic Tamarix hispida and Arabidopsis thaliana
Source: Front Plant Sci. 2021 Jan 15;11:597480. doi: 10.3389/fpls.2020.597480 (PMC7848111; doi:10.3389/fpls.2020.597480)
Supplement: Supplementary Table 1 — Classification of SOS genes in different species. [file Table_1.pdf]

TableS1. Classification of *SOS* genes in different species

Name	Species	Sequence ID
AtSOS1-1	Arabidopsis thaliana	AT2G01980
AtSOS1-2	Arabidopsis thaliana	AT1G05580
AtSOS1-3	Arabidopsis thaliana	AT1G06970
AtSOS1-4	Arabidopsis thaliana	AT1G08140
AtSOS1-5	Arabidopsis thaliana	AT1G08150
AtSOS1-6	Arabidopsis thaliana	AT1G14660
AtSOS1-7	Arabidopsis thaliana	AT1G16380
AtSOS1-8	Arabidopsis thaliana	AT1G49810
AtSOS1-9	Arabidopsis thaliana	AT1G54370
AtSOS1-10	Arabidopsis thaliana	AT1G64170
AtSOS1-11	Arabidopsis thaliana	AT1G79400
AtSOS1-12	Arabidopsis thaliana	AT1G79610
AtSOS1-13	Arabidopsis thaliana	AT2G13620
AtSOS1-14	Arabidopsis thaliana	AT2G28170
AtSOS1-15	Arabidopsis thaliana	AT2G28180
AtSOS1-16	Arabidopsis thaliana	AT2G31910
AtSOS1-17	Arabidopsis thaliana	AT3G17630
AtSOS1-18	Arabidopsis thaliana	AT3G19490
AtSOS1-19	Arabidopsis thaliana	AT3G44900
AtSOS1-20	Arabidopsis thaliana	AT3G44910
AtSOS1-21	Arabidopsis thaliana	AT3G44920
AtSOS1-22	Arabidopsis thaliana	AT3G44930
AtSOS2-1	Arabidopsis thaliana	AT1G29230
AtSOS2-2	Arabidopsis thaliana	AT1G30270
AtSOS2-3	Arabidopsis thaliana	AT1G01140
AtSOS2-4	Arabidopsis thaliana	AT1G48260
AtSOS2-5	Arabidopsis thaliana	AT2G25090
AtSOS2-6	Arabidopsis thaliana	AT2G26980
AtSOS2-7	Arabidopsis thaliana	AT2G30360
AtSOS2-8	Arabidopsis thaliana	AT2G34180
AtSOS2-9	Arabidopsis thaliana	AT3G17510
AtSOS2-10	Arabidopsis thaliana	AT2G38490
AtSOS2-11	Arabidopsis thaliana	AT3G23000
AtSOS2-12	Arabidopsis thaliana	AT4G14580
AtSOS2-13	Arabidopsis thaliana	AT4G18700
AtSOS2-14	Arabidopsis thaliana	AT4G24400
AtSOS2-15	Arabidopsis thaliana	AT4G30960
AtSOS2-16	Arabidopsis thaliana	AT5G01810
AtSOS2-17	Arabidopsis thaliana	AT5G01820
AtSOS2-18	Arabidopsis thaliana	AT5G07070
AtSOS2-19	Arabidopsis thaliana	AT5G10930
AtSOS2-20	Arabidopsis thaliana	AT5G25110

AtSOS2-21	Arabidopsis thaliana	AT5G35410
AtSOS3-1	Arabidopsis thaliana	AT5G24270
AtSOS3-3	Arabidopsis thaliana	AT4G17615
AtSOS3-5	Arabidopsis thaliana	AT4G30960
AtSOS3-6	Arabidopsis thaliana	AT4G33000
AtSOS3-7	Arabidopsis thaliana	AT5G01810
AtSOS3-8	Arabidopsis thaliana	AT5G24270
AtSOS3-10	Arabidopsis thaliana	AT5G58380
AtSOS3-12	Arabidopsis thaliana	AT4G33000
AtSOS3-13	Arabidopsis thaliana	AT5G47100
AtSOS3-14	Arabidopsis thaliana	AT1G64480
AtSOS4	Arabidopsis thaliana	AT5G37850
AtSOS4-1	Arabidopsis thaliana	AT5G37850
ATSOS5	Arabidopsis thaliana	AT3G46550
ATSOS5-1	Arabidopsis thaliana	AT3G46550
ATSOS5-2	Arabidopsis thaliana	AT3G46550
CsSOS1	Camelina sativa	XP-019087023.1
CrSOS1	Capsella rubella	XP-023633881.1
BnSOS1	Brassica napus	NP-001302668.1
BoSOS1	Brassica oleracea	VDD11642.1
BjSOS2	Brassica juncea	ABM66448.1
TahSOS2	Tarenaya hassleriana	XP-010533153.1
PtSOS3	Populus trichocarpa	XP-002318422
MnSOS3	Morus notabilis	XP-010100753.1
CsSOS4	Camelina sativa	XP-010450548.1
CsSOS4-1	Camelina sativa	XP-010450547.1
CsSOS4-2	Camelina sativa	XP-010440895.1
CsSOS4-3	Camelina sativa	XP-010435697.1
CsSOS4-4	Camelina sativa	XP-010435696.1
AnSOS4	Arabis nemorensis	VVB09126.1
CrSOS4	Capsella rubella	EOA16901.1
CrSOS4-1	Capsella rubella	XP-006284003.2
EsSOS4	Eutrema salsugineum	XP-006405823.1
BnSOS4	Brassica napus	XP-013738510.1
BnSOS4-1	Brassica napus	XP-013738509.1
BnSOS4-2	Brassica napus	XP-013742977.2
BcSOS4	Brassica cretica	RQM01982.1
BovoSOS4	Brassica oleracea var. oleracea	XP-013631976.1
BrSOS4	Brassica rapa	XP-009139754.1
CrSOS5	Capsella rubella	XP-006292833.1
AlSOS5	Arabidopsis lyrata subsp. lyrata	XP-002877492.1
CsSOS5	Camelina sativa	XP-010426092.1
CsSOS5-1	Camelina sativa	XP-019102263.1
CsSOS5-2	Camelina sativa	XP-010426091.1

CsSOS5-3	Camelina sativa	XP-010503266.1
EsSOS5	Eutrema salsugineum	XP-006418888.1
AaSOS5	Arabis alpina	KFK33988.1
AnSOS5	Arabis nemorensis	VVB11140.1
BoSOS5	Brassica oleracea	VDC97048.1
BrSOS5	Brassica rapa	XP-009149972.1
BrSOS5-1	Brassica rapa	RID58509.1
BovoSOS5	Brassica oleracea var. oleracea	XP-013630362.1
BnSOS5	Brassica napus	XP-013704484.1
BnSOS5-1	Brassica napus	XP-013642560.1
RsSOS5	Raphanus sativus	XP-018432835.1
